# Ablation of Proliferating Cells in the CNS Exacerbates Motor Neuron Disease Caused by Mutant Superoxide Dismutase

**DOI:** 10.1371/journal.pone.0034932

**Published:** 2012-04-16

**Authors:** Jean-Nicolas Audet, Geneviève Gowing, Renée Paradis, Geneviève Soucy, Jean-Pierre Julien

**Affiliations:** Research Centre of CHUQ, Department of Psychiatry and Neurosciences, Laval University, Québec, Canada; National Institutes of Health, United States of America

## Abstract

Proliferation of glia and immune cells is a common pathological feature of many neurodegenerative diseases including amyotrophic lateral sclerosis (ALS). Here, to investigate the role of proliferating cells in motor neuron disease, SOD1^G93A^ transgenic mice were treated intracerebroventicularly (ICV) with the anti-mitotic drug cytosine arabinoside (Ara-C). ICV delivery of Ara-C accelerated disease progression in SOD1^G93A^ mouse model of ALS. Ara-C treatment caused substantial decreases in the number of microglia, NG2+ progenitors, Olig2+ cells and CD3+ T cells in the lumbar spinal cord of symptomatic SOD1^G93A^ transgenic mice. Exacerbation of disease was also associated with significant alterations in the expression inflammatory molecules IL-1β, IL-6, TGF-β and the growth factor IGF-1.

## Introduction

Amyotrophic lateral sclerosis is a fatal, adult-onset and rapidly progressing neurodegenerative disorder characterized by the selective degeneration of motor neuron in the brain and spinal cord. Although the majority of ALS cases are sporadic, approximately 10% of cases are familial and dominantly inherited. Mutations in the gene encoding for the free radical-scavenging metalloenzyme, copper/zinc superoxide dismutase (SOD1) is causative in 20% of familial ALS cases [1]. Transgenic mice and rats over-expressing various ALS-related SOD1 mutants develop ALS-like phenotypes through a gain of unknown toxic properties [2]. Various mechanisms have been proposed to explain mutant SOD1 mediated motor neuron death in ALS including glutamate induced excitotoxicity, oxidative damage, ER stress, mitochondrial dysfunction, altered axonal transport and toxicity due to secreted SOD1 [3]. Moreover, it has become apparent that mutant SOD1-mediated toxicity does not occur solely within motor neurons [1], [4], [5]. Many studies have now shown that expression of mutant SOD1 in glial cells such as astrocytes and microglia causes intrinsic damage, alterations in cell function and increased cytotoxic potential of these cells which can result in enhanced damage to neighboring motor neurons [6]–[11]. Accordingly, reducing expression of mutant SOD1 in either astrocytes or microglia significantly delays disease progression and motor neuron death [4], [12] which is line with reports that wild-type microglia or astrocyte precursors promote motor neuron survival and extends lifespan of rodent models of ALS [13], [14]. Other non-neuronal cells like T lymphocytes can also confer neuroprotective inflammation in mutant SOD1 mice [15], [16].

To study the specific contribution of proliferating microglia to motor neuron degeneration, we reported previously experimental elimination of these cells by Ganciclovir treatment using doubly transgenic mice SOD1^G93A^; CD11b-TK [17]. Surprisingly, a 50% reduction in reactive microglia had no effect of motor neuron degeneration. Here, in order to achieve a more complete ablation of proliferating cells in the CNS, we tested the effects of the anti-mitotic drug cytosine arabinoside (Ara-C) in SOD1^G93A^ transgenic mice when administered intracerebroventricularly (ICV). Ara-C treatment caused efficient elimination of various proliferating cell types but with a negative impact on disease progression.

## Results

### Reduction in microglia and T cell populations in Ara-C treated SOD1^G93A^ mice

**Figure 1:**
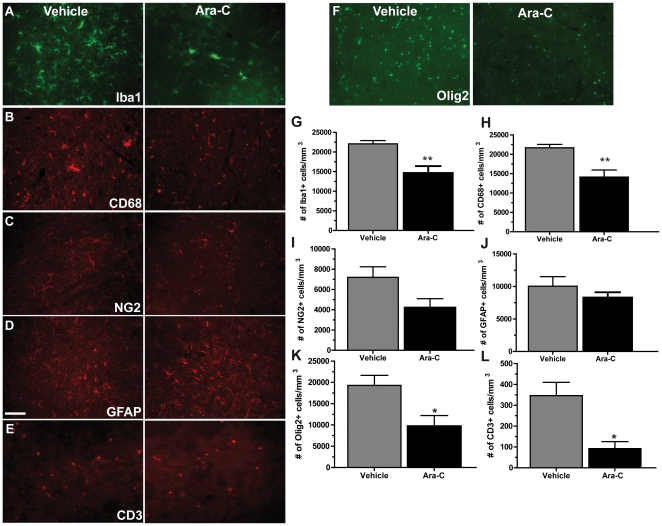
Ara-C treatment caused a decrease in microglia, NG2+ progenitors, oligodendrocytes, astrocytes and T cells in the spinal cord of SOD1^G93A^ mice. Immunofluorescence for cell markers Iba1 (A), CD68 (B), NG2 (C), GFAP (D), CD3 (E) and Olig2 (F) in the lumbar spinal cord of mutant SOD1 transgenic mice treated with vehicle or Ara-C. (G) Quantification of Iba positive cells showed a 1.5 folds reduction of cells in Ara-C treated mice compared to controls (**p = 0.0086). (H) Quantification of CD68 marker showed a 1.5 folds reduction of cells in Ara-C treated mice compared to controls (**p = 0.0099). (I) Quantification of NG2+ marker showed a 1.7 folds reduction of cells in Ara-C treated mice compared to controls (*p* = 0.0713) (J) Quantification of GFAP marked cells showed a slightly reduced number of astrocytes (1.2 folds) in Ara-C treated mice compared to controls, although this result was not significant (*p* = 0.3430). (K) Quantification of Olig2 positive cells showed a 2.0 folds reduction of cells in Ara-C treated mice compared to controls (*p = 0.0236) (L) Quantification of CD3+ cells showed 3.8 folds reduction of cells in Ara-C treated mice compared to controls (*p = 0.0122). All mice were analyzed at 115 days. All values are means ± SEM. Scale bars: 100 µm.

To determine the contribution of proliferating cells in motor neuron degeneration in ALS, we administered ICV the anti-mitotic drug Ara-C to SOD1^G93A^ transgenic mice. As proliferation of glial cells can be observed pre-symptomatically in SOD1^G93A^ transgenic mice [17], continuous ICV infusion of Ara-C was initiated at 75 days of age (pre-symptomatic) for a duration of 42 days. Here, we show that microglia, T-cells as well as NG2+ and Olig2+ cells were diminished by treatment with Ara-C (**Figure 1**). Analysis at symptomatic stage of the disease (115 days) demonstrated that treatment of SOD1^G93A^ transgenic mice with Ara-C caused a significant 1.5 folds decrease in the number of Iba1+ in the lumbar spinal cord of Ara-C treated animals compared to vehicle treated controls (p = 0.0086). Quantification of CD68+ cells, a marker for activated and phagocytic microglia, was also significantly reduced, suggesting that the ablation of activated microglia was privileged (1.5 folds, *p* = 0.0099). Although it did not reach statistical significance, a trend for a reduced number of NG2+ glial precursors was also seen (1.7 folds, *p* = 0.0713). Interestingly, a significant reduction in Olig2+ cells was observed (2 folds, *p* = 0236). No difference in the number of GFAP+ astrocytes was found (*p* = 0.3430). However, we found a significant 3.8 folds decrease in the number of CD3+ T cells in Ara-C treated mice compared to vehicle treated controls (*p* = 0.0122).

### Reduced lifespan of Ara-C treated SOD1^G93A^ mice

**Figure 2:**
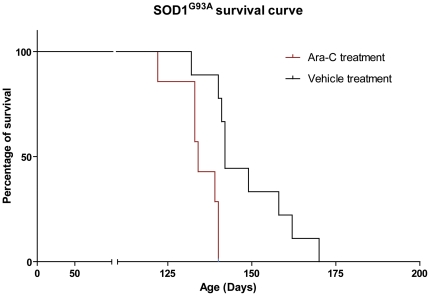
Ablation of proliferating cells in CNS decreases lifespan of SOD1^G93A^ mice. Kaplan-Meier survival curve shows that transgenic mice Sod1^G93A^ (n = 7) treated with Ara-C between 75 and 115 days had a mean survival of 134 days while untreated SOD1^G93A^ (n = 9) had a mean survival of 141 days. Log-rank test shows that this difference is significant (*p* = 0.0081).

**Figure 3:**
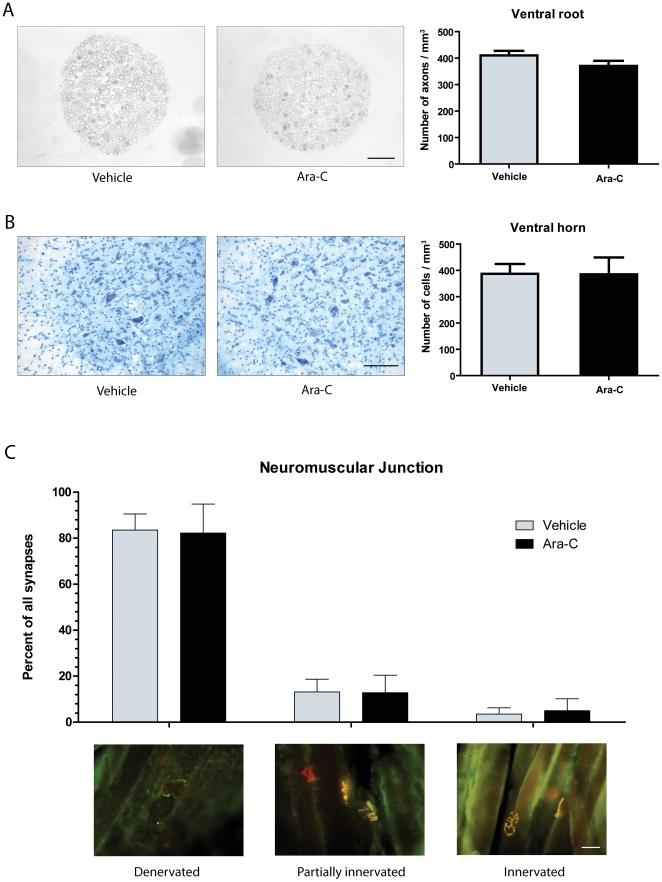
Motoneuron degeneration of SOD1^G93A^ mice was not affected by Ara-C treatment. (A) Axons in the ventral root of transgenic mice treated or with Ara-C or vehicle. Quantification of the total number of axons showed no difference between both groups (p = 0.1790). (B) Nissl stain for neuronal cells. Quantification of motoneurons showed no difference between both groups (*p* = 0.9937). (C) Immunofluorecence for innervation of muscle fibres in the gastrocnemius muscle. Number of innervated, partially innervated and denervated fibre in Ara-C treated mice did not differ from vehicle-treated controls (Denervated: *p* = 0.8326, partially innervated: *p* = 0.9281, innervated: *p* = 0.5504). (A–B) Mice were analyzed at 115 days and (C) end-stage (±140 days). All values are means ± SEM. Scale bars: (A) 100 µm and (B–C) 50 µm.

Interestingly, SOD1^G93A^ transgenic mice (n = 7) treated with Ara-C had a mean survival of 134 days while vehicle treated SOD1^G93A^ (n = 9) had a mean survival of 141 days (p = 0.0081) (**Figure 2**). However, the decreased longevity of Ara-C treated SOD1^G93A^ transgenic mice was not accompanied by an increased degeneration of the motor neuron unit. Indeed, quantification of Nissl stained motor neurons, L5 ventral root axons and neuromuscular innervations of the gastrocnemius muscle in symptomatic animals revealed no significant difference between Ara-C and vehicle treated SOD1^G93A^ transgenic mice (**Figure 3**) (motor neurons: *p* = 0.9937; axons: *p* = 0.1790; Denervated synapses: *p* = 0.8326, partially innervated synapses: *p* = 0.9281, innervated synapses: *p* = 0.5504).

### Inflammation was deregulated in Ara-C treated transgenic mice

**Figure 4:**
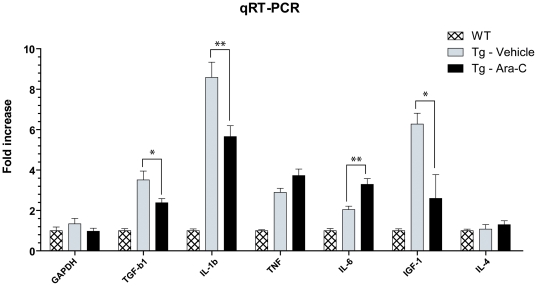
Modulation of the inflammatory response in Ara-C treated SOD1^G93A^ mice. Quantitative RT-PCR results (values are normalized to GADPH and relative to wild-type control treated with vehicle; no significant difference was found between wild-type controls treated with vehicle or M-CSF for any marker). Significant differences were found between Ara-C and vehicle treated SOD1^G93A^ in levels of mRNA for TGF-b1 (*p = 0.0148), IL-1b (**p = 0.0072), IL-6 (**p = 0.0014) and IGF-1 (*p = 0.0108). Note that all mRNA levels were significantly higher in vehicle treated transgenic mice compared to WT, treated or not (*p*<0.0025), except for IL-4 (*p* = 0.7582). All values are mean ± SEM; *n*(WT) = 9, *n*(Tg-Vehicle) = 6, *n*(Tg-Ara-C) = 15.

Real-time RT PCR analysis revealed that Ara-C induced profound changes in SOD1^G93A^ the spinal cord of mice in levels of inflammatory cytokines and growth factors (**Figure 4**). In Ara-C treated animals, the mean IL-1β mRNA level was reduced by 1.52 folds (*p* = 0.0072) compared to vehicle treated animals, which is in line with the microglia depletion. Unexpectedly, no significant changes were seen in TNF-α and IL-4 levels. In contrast, IL-6 mRNA in Ara-C treated mice was upregulated by 1.6 folds (*p* = 0.0014) whereas the growth factors TGF-β1 and IGF-1 were greatly diminished, by 1.47 (*p* = 0.0148) and 2.42 (*p* = 0.0108) folds respectively.

## Discussion

Motor neuron disease in mutant SOD1-mediated ALS is a non-cell autonomous process [1], [18]. In this study, we have shown that the depletion of proliferating cells in the CNS of SOD1^G93A^ mice, consisting mainly of NG2+ and Olig2+ cells as well as microglia and T-cells, caused a significant reduction in lifespan. Surprisingly, this decrease in survival of transgenic mice treated with Ara-C was not correlated with a reduction in motor neuron survival, axonal degeneration or muscle innervation at the symptomatic phase of disease. Similarly, we have previously observed that the specific and substantial ablation of proliferating microglia had no effect on motor neuron degeneration in mutant SOD1 mice [17]. As the assessment of motor unit degeneration was performed at the symptomatic stage of disease (115d), it is possible that the increased motor neuron degeneration that likely precipitated a reduction in survival occurred a later time in our study.

The role of oligodendrocytes in the pathology of ALS has not been extensively studied [38]. However, a study has suggested that mutant SOD1 expressing oligodendrocytes may not significantly contribute to the disease process [39]. Oligodendrocyte precursor cells express the chondroitin sulfate proteoglycan NG2, although NG2+ cells have been found to significantly proliferate in ALS we did not find a significant decrease in these cells following Ara-C treatment [17], [35]. However, it is interesting to note that a 2 fold reduction in the number of cells expressing the transcription factor Olig2 was observed. Although Olig2-positive progenitors in the embryonic spinal cord give rise can give rise to oligodendrocytes, motor neurons subset of astrocytes and ependymal cells, a considerable number of mature oligodendrocytes have been shown to be immunoreactive for Olig2 in the adult brain [36], [37]. Further study of the role of oligodendrocytes in the pathology of ALS is warranted.

*In vitro* and *in vivo* studies have shown that activated microglia can cause the degeneration of motor neurons [10], [19]–[21]. Moreover, microglia expressing mutant SOD1 have increased cytotoxic potential when compared to wild-type microglia [7], [11]. If it is correct that microglia participate in the degenerative process in ALS, why did microglia elimination by Ara-C treatment was associated with a decrease in the survival of SOD1^G93A^ transgenic mice? This may reflect the ambivalent potential of microglial cells that can be differentially activated to exhibit either neuroprotective or neurotoxic phenotypes [22]. Neurotoxic microglia express inflammatory molecules such as IL-1β, TNF-α, are a potent source of reactive oxygen species and are sometimes termed M1 microglia. On the other hand, neuroprotective microglia are characterized by the expression of anti-inflammatory cytokines and neurotrophic factors such as IGF-1. Interestingly, microglia showing a M1 neurotoxic phenotype have shown to be mostly present at the end-stages of the disease in mutant SOD1^G93A^ mice whereas the neuroprotective M2 microglia phenotype is predominantly present at the early stages of the disease [22]. In this experiment, we treated the mice at a pre-symptomatic phase of disease (75 days), a stage where the neuroprotective microglial phenotype is predominant. We may therefore have eliminated the “good” microglia, causing an adverse effect.

Treatment with Ara-C also resulted in a significant decrease in the number of CD3+ T cells in the CNS of mutant SOD1^G93A^ transgenic mice. However, whether this reduction is a direct or indirect consequence of Ara-C treatment remains unclear. Interestingly, a number of studies have shown the importance of T cells in delaying motor neuron degeneration in ALS [15], [16], [23]. However, this has been recently challenged by Tada and collegues [24]. As exacerbation of disease is paralleled with a 3.76 folds decrease in T cells, our study supports a beneficial role for these cells in SOD1 mediated ALS.

Not surprisingly, reducing the number of proliferating cells within the CNS resulted in the modulation of the inflammatory response in SOD1^G93A^ mice. However, we cannot conclude that inflammation was increased or reduced. Some pro-inflammatory cytokines were upregulated (IL-6) as well as down-regulated (IL-1β), whereas anti-inflammatory cytokines were downregulated (TGF-β1) or upregulated (IL-6). Nevertheless, a decrease in the expression of TGF-β1 and IGF-1 may have contributed to the exacerbation of disease. TGF-β is a pleitropic cytokine which regulates a wide range of cellular responses [25]. TGF-β can reduce the production of reactive oxygen species by activated microglia and promotes an M2 microglial phenotype. Interestingly, some evidence also demonstrates that TGF-β signalling can protect neurons from glutamate-mediated excitotoxicity. Moreover, the administration of TGF-β causes the transient improvement in the phenotypes of SOD1^G93A^ transgenic mice [26]. IGF-1 is another potent trophic factor produced, in part, by microglia in the CNS of SOD1 transgenic mice and has been shown to delay motor neuron degeneration in several studies using SOD1 transgenic mice or other models of motor neuron degeneration [27]–[34].

Studies investigating the role of microglial cells and inflammation in mutant SOD1 animal models have yielded confounding results. Numerous signals can influence microglial cell phenotypes and the immune responses throughout the course of the disease enabling an exacerbation of damage or protection of motor neurons. Drugs, genetic manipulation, cell transplantation or any other treatment will undoubtedly cause alterations in many signalling pathways. In depth rather and then superficial analysis of glial cell behaviour should be performed in order to reach appropriate conclusions regarding the beneficial or detrimental role of these cells in mutant SOD1-mediated ALS. For instance, the upregulation of the integrin CD11b by microglial cells is often associated with increased activation and detrimental function of microglia. Yet, in a study by Chiu and colleagues [16], a reduction in CD11b immunoreactivity by microglia in mutant SOD1 mice correlated with a decrease in IGF-1 production and in neurotrophic potential of these cells. Here, the substantial reduction in the number of microglia and T cells in the CNS of SOD1^G93A^ transgenic mice by Ara-C treatment had no effect on motor neuron survival but it significantly reduced lifespan. This supports a protective role of these cells in the pathophysiology of ALS.

## Materials and Methods

### Animals

SOD1^G93A^ transgenic mice [stock number 002726] were acquired from The Jackson Laboratory (Bar Harbor, ME). Mice were genotyped in accordance with Jackson Laboratory protocols. The use and maintenance of the mice described in this article were performed in accordance to the *Guide of Care and Use of Experimental Animals of the Canadian Council on Animal Care* (Approval number is 2011137-1).

### Surgical procedures

For intracerebroventricular delivery of Ara-C (10 mg/ml, Cytarabine; Roche) or vehicle, mice at 75 d of age were anesthetized with isoflurane and were placed in a stereotaxic apparatus (David Kopf Instruments, Tujunga, CA). The right lateral ventricule was then reached (−1.75 mm lateral, +1.00 mm antero-posterior and −2.0 mm dorsoventral to the bregma) with a 33-gauge stainless steel cannula (Plastics One, Roanoke, VA) that was connected to an Alzet osmotic mini-pump model 2004 (Durect, Cupertino, CA).

### Tissue collection for immunohistochemical analyses

At 115 days of age, following 42 days of ICV infusion of ARA-C or vehicle, mice were anesthetized and transcardially perfused with NaCl 0.9% and fixed with 4% paraformaldehyde. Another group of mice were sacrificed at end-point to assess survival but tissues were not used for analysis. Dissected spinal cord and muscle tissues were postfixed for 24 h in 4% paraformaldehyde and equilibrated in a solution of PBS-sucrose (20%) for 48 h. Spinal cord tissues were cut in 25 µm thick sections with a Leica frozen microtome and kept in a cryoprotective solution at −20°C. Gastrocnemius muscles were cut in 40 µm thick cryostat sections. Dissected dorsal root ganglia (DRG) were postfixed in a solution of 3% glutaraldehyde for a period of 48 h, washed in PBS, treated with 1% osmium tetroxide for 2 h, and dehydrated through graded alcohol solutions. Prior to Epon plastic embedding, DRG were further dissected to ensure that all ventral root axons would be sampled at a distance of 3 mm from the DRG cell body. Semithin cross-sections (1 µm) were stained with toluidine blue, rinsed, and coverslipped.

### Immunohistochemistry

Spinal cord sections were stained with the following antibodies: anti-CD68 (Serotec), anti-NG2 (Millipore), anti-GFAP (Dako or Millipore), anti-Iba1 (Wako) and anti-CD3 (BD Pharmingen) according to standard techniques. For light microscopy, sections were developed with Vectastain ABC kit, reacted with nickel-diaminobenzidine (Vector Laboratories). For immunofluorescence, sections were stained with the fluorophore-coupled secondary antibody Alexa-488, or Alexa 594 (Invitrogen) and counterstained with DAPI.

### Quantitative analyses

Every 12th section of spinal cord was immunostained for selected cellular subtype (microglia, astrocyte, glial precursors or T cells) or Nissl stained to identify motor neurons in the lumbar spinal cord. The density of labeled cells was estimated by the optical fractionator method using Stereo Investigator software (MBF Biosciences, Williston, USA) For an Iba1, CD68, GFAP, CD3, Olig2 or NG2-positive cells to be counted; a distinct cell body and visible DAPI marked nucleus had to be within the optical dissector height. The counting parameters were the distance between counting frames (400 µm), the counting frame size (100 µm×100 µm), the dissector height (13 µm) and the guard zone thickness (1 µm). Motor neurons were identified on the basis of their correct anatomical location (ventral horn/laminae 9), required a distinct nucleolus within the plane of the optical dissector and had a cross sectional area ≥250 µm^2^. Counting parameters were identical to immunostained sections except for the distance between counting frames (150 µm). Bilateral L5 ventral root axons were counted at a magnification of 60× using Stereo Investigator software and interior of axons were marked in each frame until the entire ventral root section had been sampled. Counts represent the mean axonal count for the left and right ventral roots. Bilateral gastrocnemius muscles were sampled and stained by immunofluorescence with rhodamine bungarotoxin (Invitrogen), anti-NFM (MAB5254, Millipore) and a mix of SV2 (DSHB, University of Iowa, Iowa, IA, USA) and synaptophysin (DAKO) antibodies. End-plates were scored as innervated if there was complete overlap with axon terminal, or denervated if the end-plate was not associated with an axon. Partial overlap or association with preterminal axon only was scored as intermediate innervation. Every fourth section was systematically sampled in order to evaluate all neuromuscular junctions present.

### Quantitative RT-PCR

Total RNA was extracted with Trizol reagent (Invitrogen) according to the manufacturer's instructions and then digested with deoxyribonuclease to remove any contaminating genomic DNA (Turbo DNA-free from Ambion, Austin, TX). RNA quantity and quality was assessed using an Agilent Technologies 2100 bioanalyzer and RNA 6000 Nano LabChip kit (Agilent, CA, USA). Complementary DNA (cDNA) was generated from 5625 ng of total RNA using a random primer hexamer following the protocol for Superscript II (Invitrogen). Equal amounts of cDNA (100 ng) were run in triplicate and amplified in a 15-µl reaction containing 7.5 µL of 2× Universal PCR Master Mix (Applied Biosystems, CA, USA), 10 nM of Z-tailed forward primer, 100 nM of reverse primer, 250 nM of Amplifluor Uniprimer probe (Millipore), and 1 µL of cDNA target. Moreover, no-template controls were used as recommended. The mixture was incubated at 50°C for 2 min, at 95°C for 4 min, and then cycled at 95°C for 15 s and at 55°C for 30 s 55 times using the Applied Biosystems Prism 7900 Sequence Detector. Amplification efficiencies were validated and normalized to *GADPH* gene and quantities of target gene were calculated according to a standard curve. Primers were designed using Primer Express 2.0 (Applied Biosystems). Amplicons were detected using the Amplifuor UniPrimer system were all forward primers used contained a 5′ Z sequence: ACTGAACCTGACCGTACA.

### Statistical analyses

Data were analyzed using Prism 5.0 software (Graphpad software, CA, USA). Quantification data were computed by performing two-tailed Student's *t* test and survival data using Mantel-Cox log-rank tests. Welch's correction was used when variances between samples were statistically different (determined using Fisher's test to compare variances). Data are expressed as mean ± SEM; *p*<0.05 was considered statistically significant.
